# Coarsened Exact Matching of Phaco-Trabectome to Trabectome in Phakic Patients: Lack of Additional Pressure Reduction from Phacoemulsification

**DOI:** 10.1371/journal.pone.0149384

**Published:** 2016-02-19

**Authors:** Hardik A. Parikh, Igor I. Bussel, Joel S. Schuman, Eric N. Brown, Nils A. Loewen

**Affiliations:** 1 Department of Ophthalmology, School of Medicine, University of Pittsburgh, Pittsburgh, United States of America; 2 Institute of Ophthalmology and Visual Science, Rutgers, State University of New Jersey, Newark, NJ, United States of America; 3 Department of Ophthalmology, School of Medicine, Vanderbilt University, Nashville, Tennessee, United States of America; Casey Eye Institute, UNITED STATES

## Abstract

**Purpose:**

To compare intraocular pressure (IOP) after trabectome-mediated ab interno trabeculectomy surgery in phakic patients (T) and trabectome with same session phacoemulsification (PT) using Coarsened Exact Matching. Although phacoemulsification is associated with IOP reduction when performed on its own, it is not known how much it contributes in PT.

**Methods:**

Subjects were divided into phakic T and PT. Exclusion criteria were follow-up for <12 months and additional glaucoma surgery. Demographics were compared by the Mann-Whitney U test and chi-squared test for continuous and categorical variables, respectively. Multiple imputation was utilized to avoid eliminating data with missing values. Groups were then matched using Coarsened Exact Matching based on age, race, type of glaucoma, baseline IOP, and number of preoperative glaucoma medications. Univariate linear regression was used to examine IOP reduction after surgery; those variables that were statistically significant were included in the final multivariate regression model.

**Results:**

A total of 753 cases were included (T: 255, PT: 498). When all variables except for age were kept constant, there was an additional IOP reduction of 0.05±0.01 mmHg conferred for every yearly increment in age. Every 1 mmHg increase in baseline IOP correlated to an additional IOP reduction of 0.80±0.02 mmHg. Phacoemulsification was not found to be a statistically significant contributor to IOP when comparing T and PT (p≥0.05). T had a 21% IOP reduction to 15.9±3.5 mmHg (p<0.01) while PT had an 18% reduction to 15.5±3.6 mmHg (p<0.01). Number of medications decreased (p<0.01) in both groups from 2.4±1.2 to 1.9±1.3 and from 2.3±1.1 to 1.7±1.3, respectively.

**Conclusion:**

Phacoemulsification does not make a significant contribution to postoperative IOP or number of medications when combined with trabectome surgery in phakic patients.

## Introduction

Glaucoma and cataracts often coincide in the same individual, with elevated baseline IOP resulting in a 2-fold increased odds of developing an incident nuclear cataract.[1] Although it is not established whether a true causative relationship exists between the two, several studies found that cataract surgery can lower intraocular pressure (IOP) by an average of 1.5–3 mmHg and that a higher preoperative IOP correlates with a greater postoperative IOP reduction.[2–4] Microincisional glaucoma surgeries (MIGS) can lower IOP on their own or in combination with cataract surgery.[5] Both practice patterns are standardized, safe, and can also be more cost-effective than topical medications.[6] The first studies of trabecular microbypass stents, estimated to provide access outflow segments over approximately 60 degrees,[7] demonstrated a significant IOP reduction that was similar in patients who had cataract surgery with same session microbypass stent and the control group that only had phacoemulsification.[8] Ab interno trabeculectomy with the trabectome uses plasma to ionize and remove up to 180 degrees of trabecular meshwork (TM).[5] Trabectome surgery alone or combined with phacoemulsification seemed to achieve similar postoperative IOPs in a nonrandomized comparison.[9,10] We recently applied matching to achieve a more balanced comparison of outcomes of trabectome with same session cataract surgery to cataract surgery followed by trabectome surgery years later and noted a small additive effect in IOP reduction of same session cataract removal.[11] Although statistically significant, this additional IOP reduction of 0.73±0.32 mmHg from phacoemulsification may be of limited clinical relevance.[11]

In the current study, we hypothesized that a comparison of phacoemulsification combined with trabectome surgery (PT) to trabectome (T) in eyes that remain phakic, might better isolate an IOP reduction inferred by phacoemulsification. Such a comparison is difficult to conduct without a sophisticated matching algorithm because of the inherently different demographics of individuals requiring phacoemulsification versus those that do not yet have a cataract. To precisely delineate whether same session cataract surgery had an additive IOP lowering effect without introducing confounding variables, subjects with additional glaucoma surgery were excluded. Here, we applied a recently introduced method, Coarsened Exact Matching (CEM),[12] to test our hypothesis.

## Methods

### Participants

Data for this study were collected with approval by the Institutional Review Board of the University of Pittsburgh, in accordance with the Declaration of Helsinki and the Health Insurance Portability and Accountability Act. No informed consent was necessary for this retrospective, observational cohort study. Patient records were anonymized and de-identified prior to analysis. Subjects were divided into phakic patients who received trabectome-mediated ab interno trabeculectomy (T) or combined phacoemulsification with trabectome (PT). Included were patients with a diagnosis of glaucoma with or without a visually significant cataract who had at least 12 months of follow-up with no additional glaucoma surgery. The specific target IOP was set on a case-by-case basis by the individual treating physician as the maximum IOP estimated to prevent further nerve damage. Patients with secondary open angle glaucoma (pigmentary, pseudoexfoliation, steroid-induced, and uveitic) were also included. Cases were excluded if patients were followed for less than 12 months, diagnosed with neovascular glaucoma, or were pseudophakic.

Indications for T consisted of IOP above target with worsening glaucoma on maximally tolerated topical therapy. Indications for PT were the same, or stable glaucoma with the desire to reduce the number of medications and a significant vision-impairing cataract with visual acuity testing equal to or worse than at least 0.4 logMAR (20/50 Snellen).[5,13]

Visual field status of all patients was categorized as early, moderate, or advanced by individual glaucoma specialists based on the most recent Humphrey visual field exams (Zeiss, Jena, Germany). All patients had a comprehensive slit lamp, gonioscopy, and dilated ophthalmoscopy exam prior to surgery.

### Statistics

Demographics were compared by the Mann-Whitney U test and chi-squared test for continuous and categorical variables, respectively. To avoid eliminating data with missing values, multiple imputation was utilized. Missing values of the incomplete dataset were imputed *m*>1 times, thus creating *m* completed datasets. Second, each of the *m* completed datasets were independently analyzed. Finally, the results from each of the *m* analysis were pooled into a final result. Missing data such as age, gender, and race were imputed by generating 5 similar but non-identical datasets. Groups were then matched by utilizing Coarsened Exact Matching[12] based on age, race, type of glaucoma (primary open angle glaucoma (POAG) and secondary open angle glaucoma (SOAG)), baseline IOP, and number of preoperative glaucoma medications.

Univariate linear regression was used to examine IOP reduction after surgery. Variables that were statistically significant were included in the final multivariate regression model. A p-value of less than 0.05 was considered statistically significant. Continuous variables were expressed as mean±SD. All analyses was performed using R.[14]

## Results

### Baseline Characteristics

**Fig 1:**
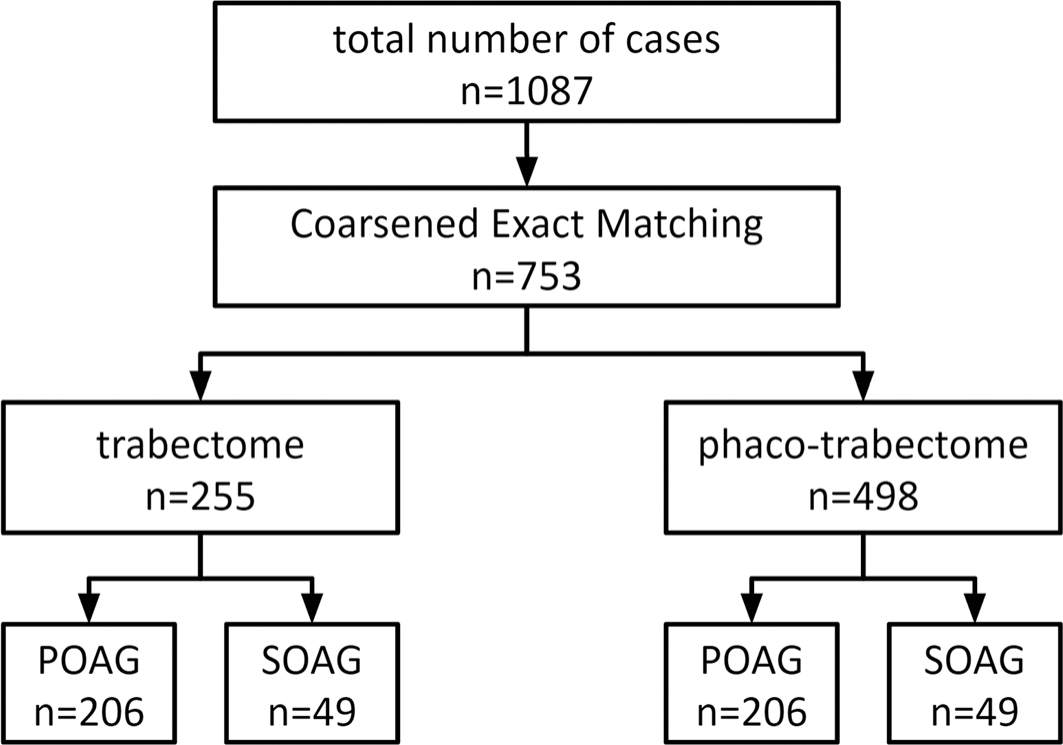
Trabectome Surgeries. Flow chart with patient assignment to trabectome and phaco-trabectome groups with stratification by glaucoma type (IOP, intraocular pressure; POAG, primary open angle glaucoma; SOAG, secondary open angle glaucoma).

After applying exclusion criteria and matching, a total of 753 cases were included in the study with 255 trabectome (T) cases and 498 phaco-trabectome (PT) cases **(Fig 1)**. Primary open angle glaucoma made up 81% and 86% of the treatment groups, respectively, with secondary open angle glaucoma making up 19% and 14%, respectively. Baseline demographics of both groups are shown in **Table 1**.

**Table 1:** Raw Demographics. Demographics for Trabectome and Phaco-Trabectome show significant differences (p<0.05) for age, race, baseline IOP, and baseline number of medications in the unmatched data.

	Trabectome (n = 445)	Phaco-Trabectome (n = 642)	p-value
Age			<0.01*
Mean±SD	63±15	73±9	
Range	(18, 96)	(27, 94)	
Type			0.15
Angle closure	4 (1%)	10 (2%)	
POAG	324 (73%)	486 (76%)	
SOAG	111 (25%)	130 (20%)	
Race			<0.01*
African American	34 (8%)	29 (5%)	
Asian	119 (27%)	206 (32%)	
Caucasian	216 (49%)	328 (51%)	
Other	52 (12%)	20 (3%)	
Baseline IOP			<0.01*
Mean±SD	24.7±8.0	20.7±6.4	
Range	(10, 56)	(10, 59)	
Baseline # of Meds			<0.01*
Mean±SD	2.8±1.4	2.3±1.2	
Range	(0, 7)	(0, 5)	

*The means are not weighted

Age (p<0.01), race (p<0.01), baseline number of glaucoma medications (p<0.01), and baseline IOP (p = <0.01) were found to be statistically different between groups. After Coarsened Exact Matching was applied, preoperative differences between the treatment groups were minimized **(Table 2)**. Age and race were now much more similar but differences still remained significant (p<0.03).

**Table 2:** Matched Demographics. Matched data for the Trabectome and Phaco-Trabectome groups were highly similar.

	Trabectome (n = 255)	Phaco-Trabectome (n = 498)	p-value
Age			<0.01*
Mean±SD	69±11	72±9	
Range	(31, 95)	(36, 93)	
Type			0.06
Angle closure	0 (0%)	0 (0%)	
POAG	206 (81%)	430 (86%)	
SOAG	49 (19%)	68 (14%)	
Pigmentary	13	13	
Pseudoexfoliation	28	45	
Steroid-induced	2	3	
Uveitic	1	0	
Unspecified	5	7	
Race			0.03*
African American	13 (5%)	23 (5%)	
Asian	68 (27%)	159 (32%)	
Caucasian	162 (64%)	309 (62%)	
Other	12 (5%)	7 (1%)	
Baseline IOP			<0.01*
Mean±SD	21.7±7.0	19.7±5.8	
Range	(10, 56)	(10, 59)	
Baseline # of Meds			0.05
Mean±SD	2.4±1.2	2.3±1.1	
Range	(0, 5)	(0, 5)	

*The means are not weighted

### Multiple Imputation and Coarsened Exact Matching

Missing data in each category were recorded. Glaucoma type and race had missing data among both groups. T had an unknown glaucoma type in 1% and unknown race in 4% of patients. PT had an unknown glaucoma type in 2% and unknown race in 9% of patients. These missing values were imputed by generating 5 similar but non-identical completed datasets. Each completed dataset was then matched to minimize the baseline differences between treatment groups.

### Linear Regression Models

A linear regression of the multiple imputed, matched data was used to identify the magnitude of various parameters on the IOP lowering effect of surgery. Univariate linear regression was first performed **(Table 3)** using the variables age, race, phacoemulsification, SOAG (secondary open angle glaucoma), baseline IOP, and number of medications at baseline.

**Table 3:** Univariate Regression Results. Age, phacoemulsification, SOAG, baseline IOP, and baseline number of medications were statistically significant (p<0.05).

	Coefficient	Std. Error	p-value
Phaco	-1.75	0.5	<0.01*
Age	0.06	0.03	0.03*
Race			
Asian	0.94	1.22	0.44
Caucasian	0.28	1.16	0.81
Other	3.24	1.91	0.09
SOAG	5.03	0.61	<0.01*
Baseline IOP	0.82	0.02	<0.01*
Baseline Rx	0.59	0.21	0.01*

*The means are not weighted

Of these, all except race were found to be statistically significant and were included in the final multivariate regression model **(Table 4)**. Age, SOAG, and baseline IOP were found to be statistically significant in both models (p<0.03, p<0.01, and p<0.01, respectively).

**Table 4:** Multivariate Linear Regression. Results for patient parameters that were found to be statistically significant (p<0.05) in univariate linear regression.

	Coefficient	Std. Error	p-value
Intercept	-14.78	1.18	<0.01*
Phaco	-0.21	0.28	0.46
Age	0.05	0.01	<0.01*
SOAG	1.21	0.38	<0.01*
Baseline IOP	0.8	0.02	<0.01*
Baseline # of Meds	-0.07	0.12	0.54

*The means are not weighted

**Fig 2:**
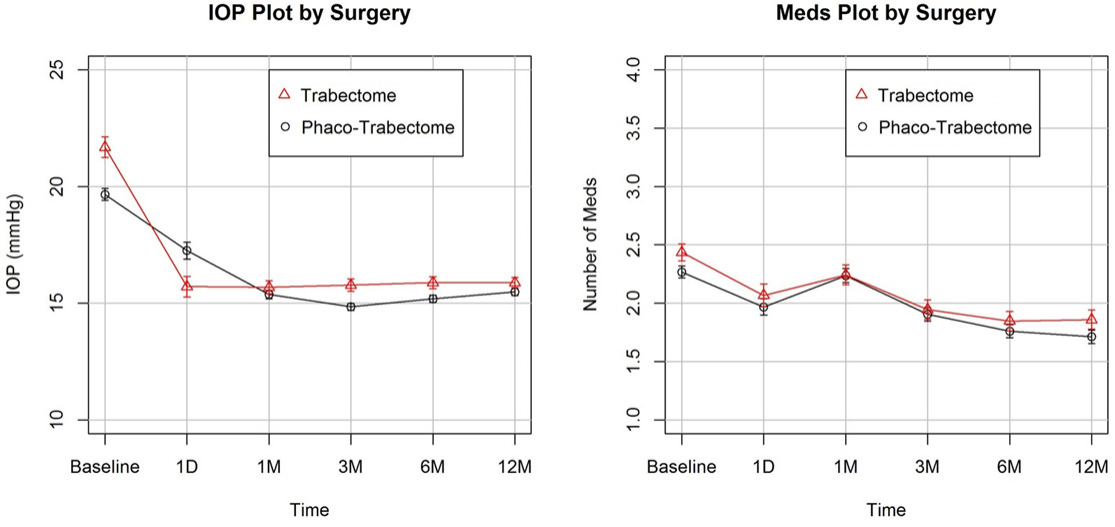
IOP and Medication Plots. IOP outcomes plot (left) demonstrates statistically significant (p<0.01) reductions in both groups (T: 21%, PT: 18%) at 12 months postoperatively. The Number of Medications plot (right) also demonstrates significant (p<0.01) decreased by 19% and 17% in each group, respectively.

When all variables except for age were kept constant, there was an additional mean IOP reduction of 0.05±0.01 mmHg conferred for every one unit increment in age. Similarly, SOAG patients had a significant IOP reduction of 1.21±0.38 mmHg more than POAG patients. Finally, every 1 mmHg increment in baseline IOP correlated to an additional IOP reduction of 0.80±0.02 mmHg. Principally, phacoemulsification was not found to be a statistically significant factor (p≥0.05) in IOP outcomes between patients who underwent T and PT (**Fig 2**). Trabectome-only patients had a 21% reduction in IOP to 15.9±3.5 mmHg (p<0.01) at 12 months postoperatively while phaco-trabectome patients underwent an 18% reduction to 15.5±3.6 mmHg (p<0.01). Number of medications also significantly decreased (p<0.01) in both groups to 1.9±1.3 (19% reduction) and 1.7±1.3 (24% reduction), respectively.

**Fig 3:**
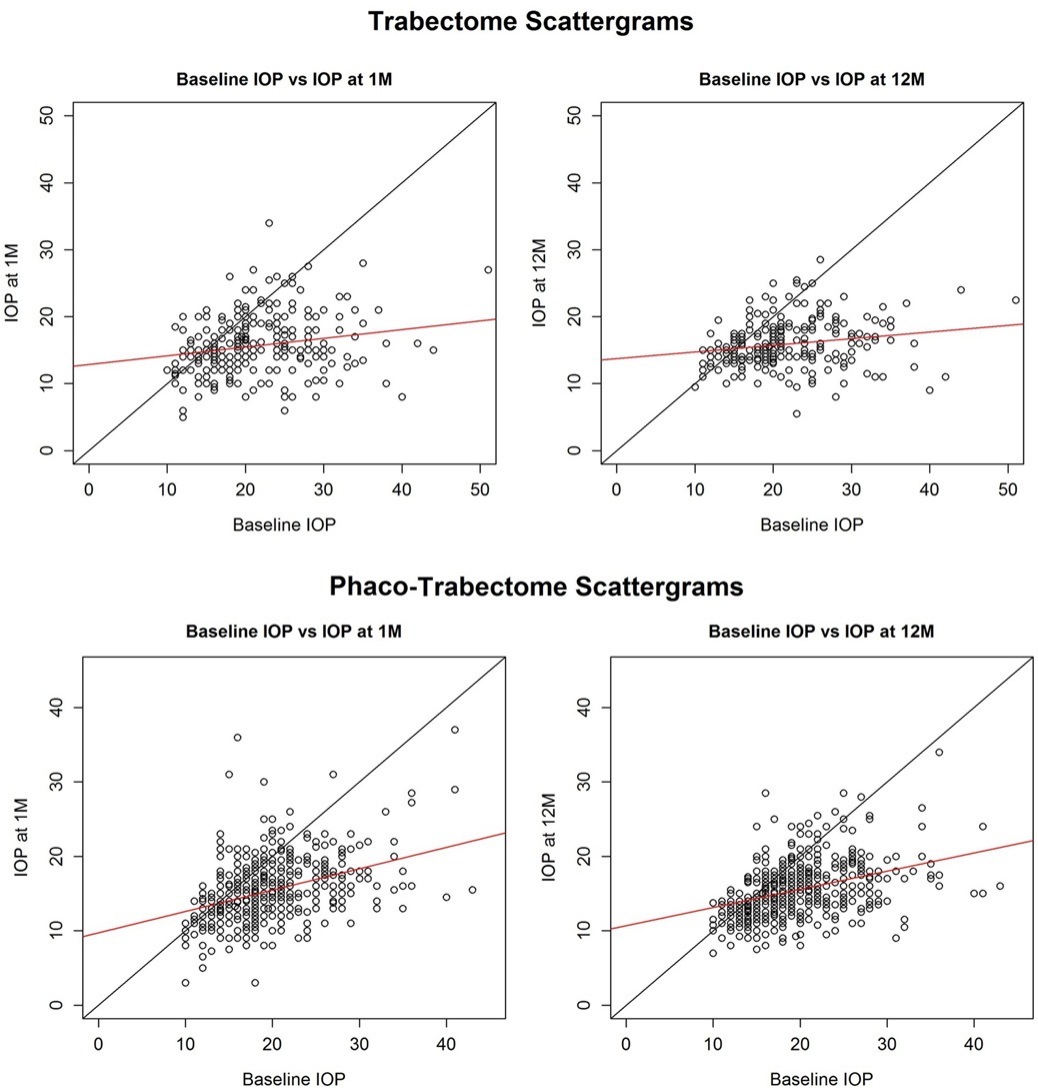
Intraocular Pressure Scattergrams. Scattergrams demonstrating IOP outcomes in comparison to baseline values among the Trabectome and Phaco-Trabectome cohorts at the 1 and 12 month postoperative visits (red line = linear fit). Circles below the black dividing line are eyes with a postoperative IOP that was lower than the preoperative IOP at that visit.

Scattergrams were generated to compare baseline IOP to IOP at 1 month and 12 months between the T and PT cohorts (**Fig 3)**. The black line indicates equal pre- and postoperative IOP and eyes with a successfully lowered IOP would be expected below this line. Most eyes had a postoperative IOP at the visit shown that was lower than the preoperative IOP. The red line is a linear fit of pre- and postoperative IOPs.

## Discussion

Using Coarsened Exact Matching, we found that phacoemulsification does not make a significant difference in postoperative IOP when combined with trabectome surgery. Both groups achieved a substantial reduction in IOP and number of medications. Hence, no additional IOP reduction should be expected from combining phacoemulsification with trabectome surgery when compared to a patient with similar baseline characteristics who received trabectome surgery alone and remains phakic. This holds true despite the mixed indication in PT because the medications were reduced equally in both T and PT. The indication in PT consisted of a visually significant cataract plus uncontrolled glaucoma or a visually significant cataract and the desire to reduce medications in glaucoma that is otherwise controlled.

The raw data age differences between the T and PT group are consistent with the increased incidence of cataracts with age.[15] Race, baseline IOP, and number of medications at baseline were also significantly different in both cohorts and may represent glaucomas with a more aggressive phenotype. After applying multiple imputation and Coarsened Exact Matching, missing data was filled in as to not distort any relationships contained in the data while enabling the inclusion of all observed data.[16] The differences in the groups were minimized and allowed for a statistically valid comparison with linear regression. Earlier studies had assumed that IOP reduction after trabectome surgery were independent of preoperative IOP[10,17] and limited by episcleral venous pressure. This data suggests that elements downstream of the TM contribute to the outflow resistance. This is consistent with results of excimer laser ablation towards Schlemm’s canal from ab externo that found that a significant reduction closer to the outer wall of Schlemm’s canal.[18]

Our results indicate that the IOP reduction around 1.5 to 3 mmHg seen after phacoemulsification[2–4] is likely mediated by the TM. The partial bypassing of TM when using microstents[7] compared to the more extensive TM removal[5] may make such an effect of phacoemulsification on TM more noticeable. Although a recent study has suggested that phacoemulsification and intraocular lens implantation alone may be effective in decreasing IOP in glaucomatous eyes,[19] this effect is often unreliable.[20] In contrast, trabectome-mediated ab interno trabeculectomy in our study achieved a profound IOP reduction already on day 1 and week 1 without any of the dangerous IOP elevations that can be seen in eyes with glaucoma undergoing cataract surgery.[21] Thus, unlike studies that have shown synergistic IOP reduction when combining a microbypass stent implantation with cataract removal,[8,22,23] the results here suggest that adding phacoemulsification to trabectome surgery in hopes of further reducing pressure may not provide these additional benefits. It may be more cost effective and safer to only focus on TM ablation in patients without a visually significant cataract.

Limitations of this study are inherent to the observational nature compared to randomized controlled trials. However, the coarsened exact matching (CEM) used here and multiple imputation avoids discarding valid data. Although observational data is easy to collect compared to randomized experiments, the mechanism of treatment assignment and other aspects of data generation are often ambiguous and difficult to control. CEM is a newer form of automatic, nonparametric matching to control the confounding influence of pretreatment control variables by achieving an acceptable balance between treated and control groups. CEM belongs to the class of Monotonic Imbalance Bounding (MIB) that requires no assumptions about the data generation process.[12]

In conclusion, we use coarsened exact matching to achieve a balanced comparison between two unequal treatment groups, trabectome-mediated ab interno trabeculectomy combined with phacoemulsification and trabectome-mediated ab interno trabeculectomy in phakic patients. We find that phacoemulsification does not contribute significantly to IOP reduction in these patients.
